# Low-intensity pulsed ultrasound in combination with SonoVue induces cytotoxicity of human renal glomerular endothelial cells via repression of the ERK1/2 signaling pathway

**DOI:** 10.1080/0886022X.2018.1487868

**Published:** 2018-08-20

**Authors:** Xiu Liu, Bei Wang, Hongyu Ding, Hao Shi, Ju Liu, Hongjun Sun

**Affiliations:** a Shandong Provincial Qianfoshan Hospital, Shandong University, Jinan, China;; b Department of Cardiography, Yantai Affiliated Hospital of Binzhou Medical University, Yantai, China;; c Department of Ultrasonography, Shandong Provincial Qianfoshan Hospital, Shandong University, Jinan,China;; d Department of Radiology, Shandong Provincial Qianfoshan Hospital, Shandong University, Jinan, China;; e Medical Research Center, Shandong Provincial Qianfoshan Hospital, Shandong University, Jinan, China

**Keywords:** Low intensity pulsed ultrasound, SonoVue, cytotoxicity, human renal glomerular endothelial cells, ERK1/2 signaling pathway

## Abstract

**Objectives:** Low-intensity pulsed ultrasound (LIPUS) and SonoVue have been used widely for diagnosis and therapeutic treatment. The effects of LIPUS and SonoVue on the microvascular system and underlying molecular mechanisms have not been established.

**Methods:** Cultured human renal glomerular endothelial cells (HRGECs) were treated with 5-min ultrasonic irradiation, 20% SonoVue or the combination of both treatments. Cell proliferation, viablity, and apoptosis were measured by MTT assay, Trypan blue exclusion assay and flow cytometry, respectively. Activation of extracellular regulated protein kinases (ERK) were examined by Western blot.

**Results:** We found that LIPUS and SonoVue alone do not induce cytotoxicity of HRGECs; however, the combination of the two treatments reduces cell proliferation and increases cell death. In addition, the combination of LIPUS and SonoVue suppressed the activation of ERK 1/2 in HRGRCs. With pretreatment of the inhibitor of ERK1/2 signaling, PD98059, LIPUS, and SonoVue does not induce additional cell death and inhibition of proliferation.

**Conclusions:** LIPUS combined with SonoVue induces cytotoxicity of HRGECs via repression of the ERK1/2 signaling pathway.

## Introduction

Low-intensity pulsed ultrasound (LIPUS) refers to lower intensity at the range of 0–0.5 W/cm^2^ than traditional ultrasound with a form of pulse wave [1]. LIPUS induces both thermal and non-thermal effects [2]. The thermal effect is negligible owing to its lower intensity and pulsed mode, while the non-thermal effect may have therapeutic effects in various tissues [3]. The non-thermal effect of LIPUS includes cavitation, microstreaming, and micromassage [4]. Cavitation contributes mainly to the therapeutic application of LIPUS [5]. As cavitation causes physical damage to the cell membrane, LIPUS affects various biological processes such as DNA and protein synthesis [6,7].

Ultrasound contrast agents are a series of microbubbles, which are composed of gas cores encapsulated by lipid membranes [8]. The SonoVue agent is a brand of the most commonly used microbubbles. When microbubbles are injected intravenously and enter the blood circulation, the microbubbles oscillations induced by ultrasound irradiation interact with vasculature [9]. The inner wall of the blood vessels, composed by a single layer of endothelial cells, are directly affected by microbubbles in the blood circulation. Endothelial cells show markedly heterogeneity in morphology and functions [10]. The treatment of LIPUS and ultrasound contrast agent might induce distinct effects on the specific subsets of endothelial cells. However, the vascular bed-specific effects of microbubbles on endothelial cells have not been investigated.

HRGECs are an unique subtype of endothelial cells, which constitute the renal glomerulus filter barrier in combination with podocytes and the glomerular basement membrane [11,12]. HRGECs contain numerous fenestrae, which are of the width ranging from 60 to 100 nm and cover around 30–40% of the endothelial cell surface as efficient portals [13]. This structure allows the high water permeability [14]. In addition, the surface of HRGECs is covered by glycocalyx, a structure made of proteogycans, glycoproteins, and sialic acids. Due to the effects of glycocalyx, fenestrated HRGECs are not as permeable to water as to macromolecules [13]. The structure of HRGECs might be modified by the environmental stresses and cause malfunctions of the glomerulus filter barrier [11,15].

The mitogen-activated protein kinases (MAPK) signal pathways play a vital role in mediating a wide range of extracellular stimuli on cellular functions [16]. MAPK cascade contains three modules: extracellular signal-regulated kinase (ERK), p38 MAPK and c-Jun N-terminal kinase (JNK). The ERK module is composed of Raf, MEK1/2 (MAPKK), and ERK1/2. ERK1/2 regulates proliferation, differentiation and apoptosis in a variety of cell types [17,18]. Recent studies indicated that LIPUS might activate ERK1/2 signaling pathway in synovial cells and human foreskin fibroblasts [19,20]. However, the effects of SonoVue or LIPUS combined with SonoVue on ERK1/2 signaling are still unknown.

In this study, the effects of LIPUS and SonoVue microbubbles on HRGECs were examined. We found that LIPUS or SonoVue alone did not affect HRGECs proliferation, viability, and apoptosis. However, LIPUS combined with SonoVue, which mimics microbubble-assisted ultrasound, induced a decrease of proliferation and viability, and an increase of apoptosis of HRGECs. In addition, our data demonstrated that the cytotoxic effects of LIPUS combined with SonoVue are mediated by the suppression of the ERK1/2 signaling pathway.

## Materials and methods

### Ultrasonic device and ultrasound contrast agents

The experiments were performed by US 13 dual-band ultrasonic therapeutic apparatus (Cosmogamma, Emildue, Italy) with a digital timer, duty cycle regulator, and intensity controller. The duty cycle (DC) ranged from 10% to 90%, while the intensity varied from 0.1 to 1.5 w/cm^2^ at a growth rate of 0.1 w/cm^2^ with a stationary pulse repetition frequency of 100 Hz [21]. The procedure used in this study was 1 MHz, 0.3 w/cm^2^, 20% DC, 5 min. The SonoVue agent (Bracco, Milan, Italy) was prepared following the manufacturer’s instructions. The lyophilized power and sulfur hexafluoride gas (SF6) in a bottle was rapidly filled with 5 ml of 0.9% normal saline solution. Then the bottle was vigorously vibrated to produce microbubbles until lyophilized power was dispersed completely.

### Cell culture

HRGECs were obtained from ScienCell Research Laboratories (Carlsbad, CA, USA), and cultured in DMEM (Gibco; ThermoFisher Scientific, Grand Island, NY, USA) supplemented with 10% fetal bovine serum (ThermoFisher Scientific), 100 IU/ml penicillin and 100 μg/ml streptomycin (ThermoFisher Scientific). In the experiments, cells were trypsinized (Millipore, Billerica, MA, USA), resuspended and counted for a density of 2–5 × 10^6^ cells/ml. Then the single-cell suspension was added into 3-ml polystyrene flat tubes. Each tube contained 1 ml of cell suspension of HRGECs. PD90859 (Cell Signaling Technology, Danver, MA, USA) was dissolved in dimethyl sulfoxide, and the pretreatment was defined as 10 μm PD98059 for 1 h before the exposure of LIPUS or SonoVue [12]. Cells were divided into four treatment groups: Control group (C); LIPUS group (U); SonoVue group (S); LIPUS and SonoVue group (U + S).

### Ultrasonic device and ultrasound contrast agents

The experiments were performed by US 13 dual-band ultrasonic therapeutic apparatus (Cosmogamma, Emildue, Italy) with a digital timer, duty cycle regulator, and intensity controller. The duty cycle (DC) ranged from 10% to 90%, while the intensity varied from 0.1 to 1.5 w/cm^2^ at a growth rate of 0.1 w/cm^2^ with a stationary pulse repetition frequency of 100 Hz [21]. The procedure used in this study was 1 MHz, 0.3 w/cm^2^, 20% DC, 5 min. The SonoVue agent (Bracco, Milan, Italy) was prepared following the manufacturer’s instructions. The lyophilized power and sulfur hexafluoride gas (SF6) in a bottle was rapidly filled with 5 ml of 0.9% normal saline solution. Then the bottle was vigorously vibrated to produce microbubbles until lyophilized power was dispersed completely.

### Cell proliferation and viability

The proliferation of HRGECs was assessed by a MTT assay kit (Solarbio, Beijing, China). Cells were seeded at 5000/well on a 96-well plate. At 12 and 24 h after treatment, the cells were washed with PBS and incubated with 10 μl of MTT solution in each well for 4 h. Then 110 μl formazan dissolving solution was added to each well to solubilize the crystals. The colorimetric intensity was measured at 490 nm by a plate reader (Molecular Devices, Sunnyvale, CA, USA). Trypan blue exclusion assay were performed to determine the viability of HRGECs. Cells were washed by PBS and incubated in 0.05% trypsin at 37 °C for 5 min. After dispersing, the single-cell suspension was diluted by 9:1 in 0.4% trypan blue (Solarbio, Beijing, China). Using a hemocytometer, the stained and stain-free cells were counted under an optical microscope.

### Cell apoptosis

At 24 h after treatments, the apoptosis of HRGECs was assessed by an Annexin V-FITC and Propidium iodide (PI) staining kit (Neobiosciences, Shenzhen, China) following the manufacturer’s instructions. Briefly, the cells were trypsinized, washed and resuspended into single cells in binding buffer. Then the cells were stained with 5 μl of 0.25% Annexin V-FITC for 3 min and 10 μl of 1 μg/ml PI for 10 min in dark at room temperature. The staining was detected by a flow cytometer (BD FACSAria II; BD Biosciences, San Jose, CA, USA) at an excitation wavelength of 490 nm. Data collection and processing was performed with the FACSDiva software (BD Biosciences).

### Western blotting

HRGECs were washed and then lysed with standard RIPA buffer (Solarbio) supplemented with PMSF 1 mM PMSF, 10 μg/ml leupeptin, and 1 μg/ml protease inhibitors aprotonin. The protein concentration of the cell lysate was determined by BCA assay (Bio-Rad, Hercules, CA, USA). The protein samples were electrophoresed through an 8% polyacrylamide gel, which was then transferred to a polybinylidene fluoride membrane. After blocking in TBST with 5% nonfat milk in for 2 h at room temperature, the membrane was incubated overnight at 4 °C with primary antibodies in the antibody diluents (Beyotime, Jiangsu, China). The primary antibodies were p44/42 MAPK (ERK1/2) (137F5), phospho-p44/42 MAPK (ERK1/2) (D13.14.4E), and anti-GAPDH (14C10) (Cell Signaling Technology). The secondary antibody was goat anti-rabbit IgG conjugated with peroxidase (BA1054) (Boster, Wuhan, China). The immunoblots were visualized by enhanced chemiluminescence (ECL) reagents (Millipore). Densitometry analyses of the blots were performed with Image-Pro Plus 6.0 software (Media Cybernetics, Rockville, MD, USA).

### Statistical analysis

Statistics analyses were performed with SPSS 19.0 software (SPSS Inc., Chicago, IL, USA). Data were presented as the means ± standard deviation (SD). Statistical differences between the groups were examined using one-way ANOVA and Student *t*-test. A *p* values less than .05 was considered statistically significant in all comparisons.

## Results

### LIPUS combined with SonoVue induces cytotoxicity on HRGECs

**Figure 1.**
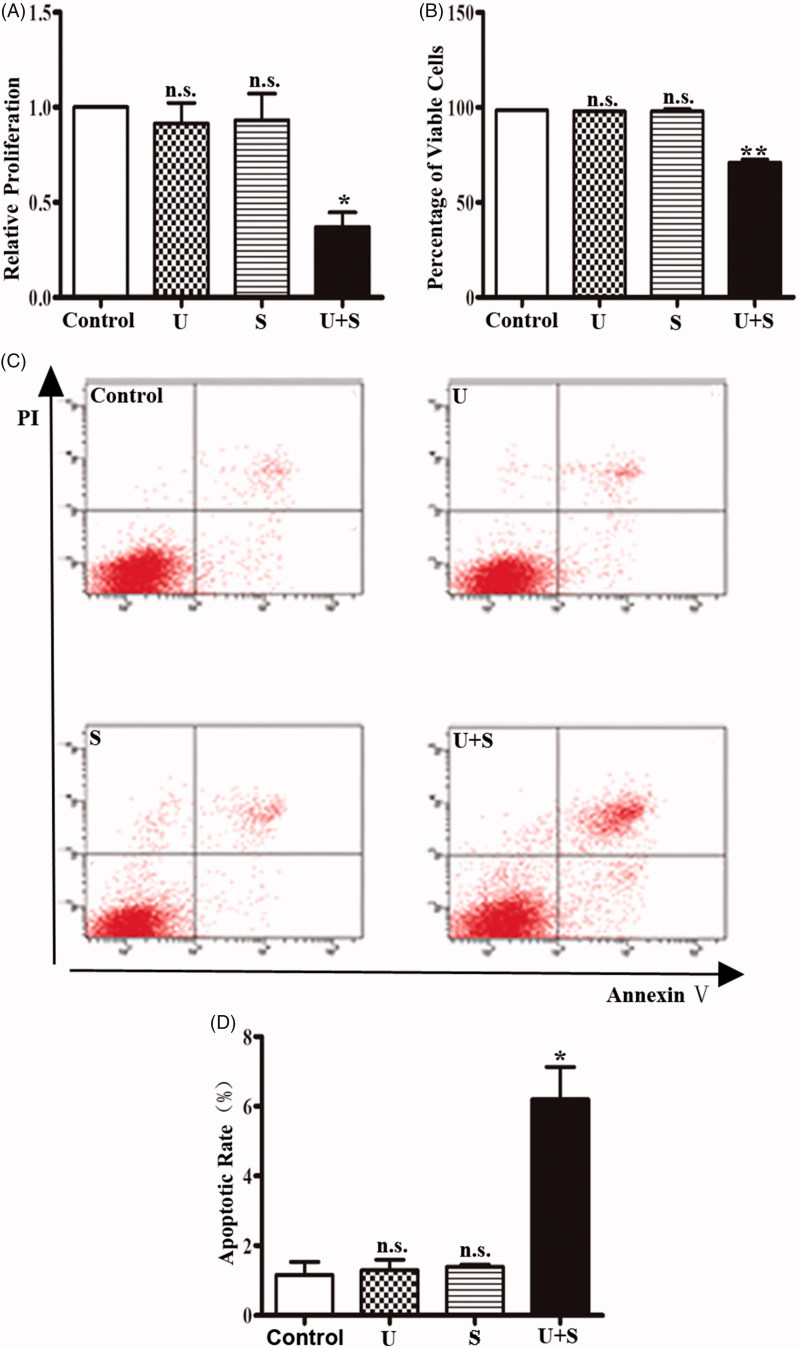
The effects of LIPUS and SonoVue on proliferation, viability and apoptosis of HRGEC. (A) MTT assay of HRGECs treated with LIPUS, SonoVue, or LIPUS combined with SonoVue. The optical density reading was converted to relative cell number. *n* = 4. n.s., non-significant. *p < .05; (B) Trypan blue exclusion of assay of HRGECs treated with LIPUS, SonoVue, or LIPUS combined with SonoVue. *n* = 4. n.s., non-significant. **p < .01; (C) Representative images of flow cytometry analyses of Annexin V/PI double staining for HRGECs with treated with LIPUS, SonoVue, or LIPUS combined with SonoVue, following by incubation with fresh media for 24 h. (D) Analyses of the apoptosis rate obtained from Annexin V-FITC/PI assay. *n* = 4. n.s., non-significant. *p < .05; U: LIPUS; S: SonoVue; U + S: LIPUS and SonoVue.

The proliferation of HRGECs treated with LIPUS and SonoVue was examined by MTT assay. Figure 1(A) indicated that the relative absorbance of the control group, LIPUS group and the SonoVue group was similar, but the LIPUS combined with SonoVue group was significant lower than the other groups (*p* < .05). To evaluate the cell viability, the cells were detected by trypan blue exclusion assay immediately after different treatments. Similarly, cell viability was significantly lower in the LIPUS combined with SonoVue group than the other groups (Figure 1(B)) (*p* < .01), while the LIPUS or SonoVue alone showed no significant effect on the viability of HRGECs. In addition, apoptosis of HRGECs were examined by Annexin V/PI staining following flow cytometry analysis. After treatments with LIPUS and SonoVue, HRGECs were cultured for 24 h in the 6-well plate before collection for flow cytometry. As shown in Figure 1(C and D), apoptotic rate was increased in HRGECs treated LIPUS combined with SonoVue (*p* < .05) while not in the HRGECs treated with LIPUS (*p* = .794) or SonoVue (*p* = .595) alone. Thus, LIPUS combined with SonoVue decreases proliferation and increases cell death in HRGECs.

### LIPUS combined with SonoVue inhibits ERK1/2 activation in HRGECs

**Figure 2.**
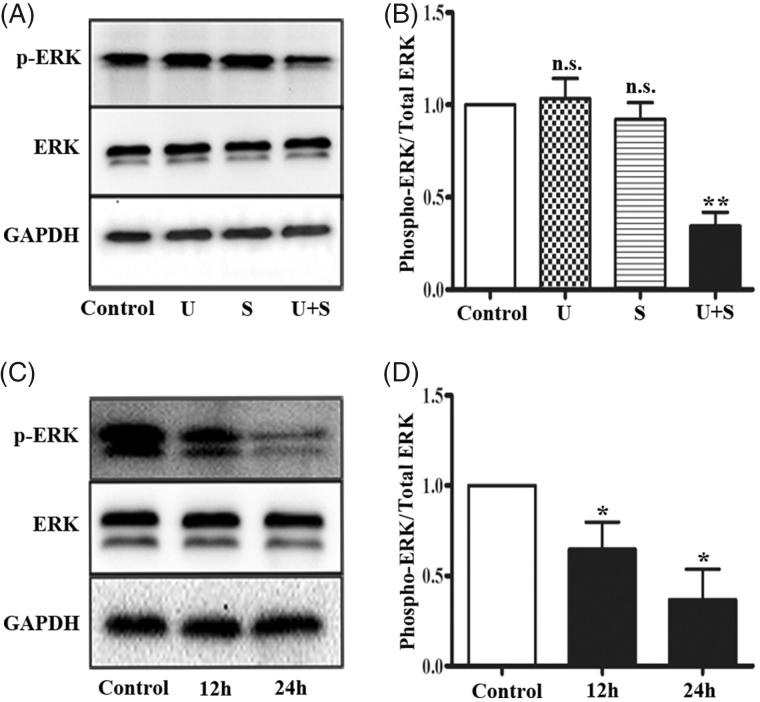
LIPUS in combination with SonoVue decreases phosphorylation of ERK1/2 in HRGECs. (A) Representative blots of phospho-ERK1/2 and total ERK1/2 from samples of HRGECs treated with LIPUS, SonoVue, or LIPUS combined with SonoVue. (B) Densitometry analyses of phospho-ERK1/2/total ERK1/2 of (A). *n* = 4. n.s., non-significant. **p < .01; (C) Representative blots of phosphor-ERK and total ERK from samples of HRGECs treated with LIPUS combined with SonoVue for 12 and 24 h after treatments. (D) Densitometry analyses of phospho-ERK1/2/total ERK1/2 of (A). *n* = 4, *p < .05; U: LIPUS; S: SonoVue; U + S: LIPUS; and SonoVue. GAPDH was used as loading control.

In response to environmental stresses, ERK1/2 are activated by MAPK/ERK kinase (MEK) 1 and MERK2, which phosphorylate the Thy-Glu-Tyr motif [22]. By Western blotting, we examined the effects of LIPUS and SonoVue on ERK1/2 signaling in HRGECs. As shown in Figure 2(A and B), phosphorylation of ERK1/2 did not change in HRGECs treated with LIPUS (*p* = .886) or SonoVue (*p* = .637) alone, but significantly decreased in HRGECs treated with LIPUS and SonoVue together (*p* < .01). The total ERK protein level was similar in all four groups. Then we performed a time course study on the HRGECs treated with LIPUS combined with SonoVue, and found that phospho-ERK1/2 was significantly decreased at both 12 h (*p* < .05) and 24 h (*p* < .05) (Figure 2(C and D)). Thus, ERK1/2 signaling was inhibited in HRGECs by the combined effects of LIPUS and SonoVue.

### With pretreatment of PD98059, LIPUS and SonoVue did not induce additional cytotoxic effects

**Figure 3.**
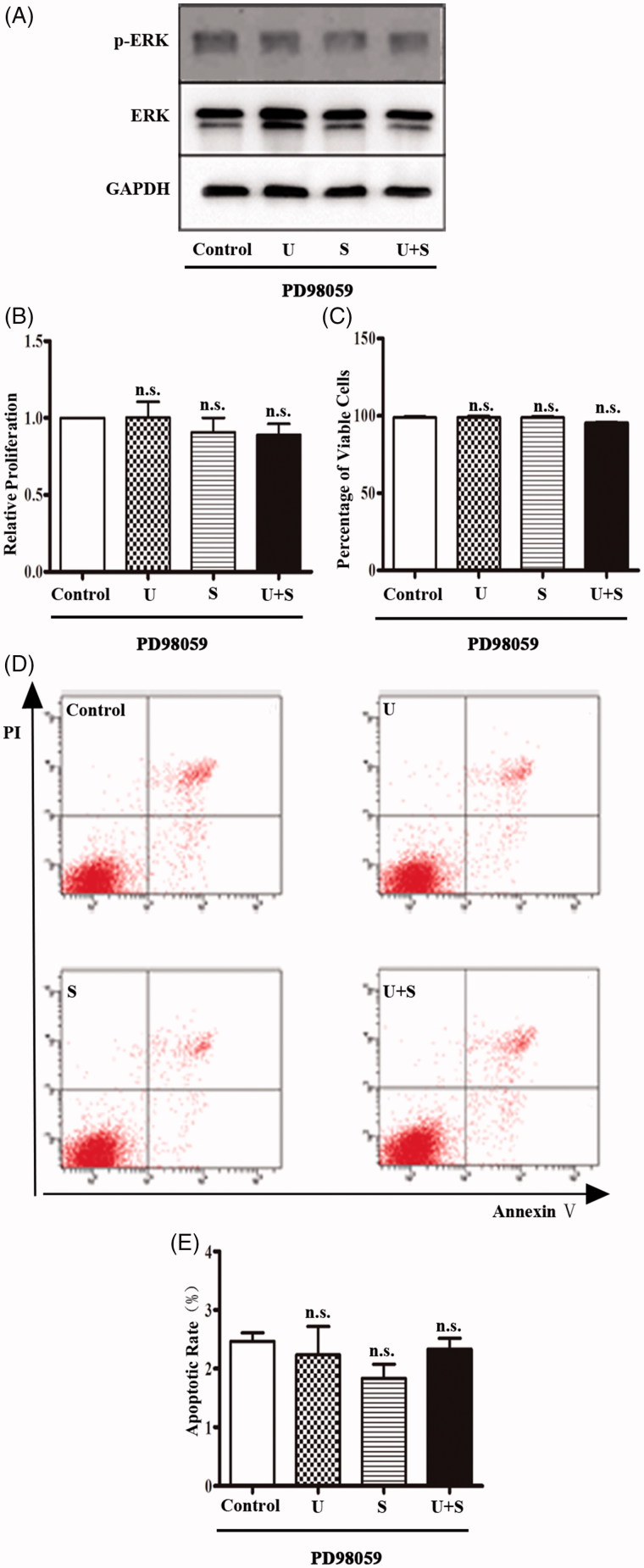
The effects of LIPUS and SonoVue on proliferation, viability, and apoptosis of HRGEC pretreated with PD98059. (A) Representative blots of phospho-ERK1/2 and total ERK1/2 from samples of HRGECs treated with LIPUS, SonoVue, or LIPUS combined with SonoVue after 1 h pretreatment of PD98059. GAPDH was used as loading control. (B) MTT assay of HRGECs treated with LIPUS, SonoVue, or LIPUS combined with SonoVue after 1 h pretreatment of PD98059. The optical density reading was converted to relative cell number. *n* = 4. n.s., non-significant; (C) Trypan blue exclusion of assay of HRGECs treated with LIPUS, SonoVue, or LIPUS combined with SonoVue after 1 h pretreatment of PD98059. *n* = 4. n.s., non-significant; (D) Representative images of flow cytometry analyses of Annexin V/PI double staining for HRGECs with treated with LIPUS, SonoVue, or LIPUS combined with SonoVue after 1 h pretreatment of PD98059, following by incubation with fresh media for 24 h. (E) Analyses of the apoptosis rate obtained from Annexin V-FITC/PI assay. *n* = 4. n.s., non-significant; U: LIPUS; S: SonoVue; U + S: LIPUS and SonoVue.

PD98059 is a selective inhibitor of ERK1/2 signaling pathway by combining and devitalizing MEK [23]. HRGECs were pretreated with 10 μM of PD98059 for 1 h before LIPUS and/or SonoVuetreatments [12]. After pretreatment with PD98059, the level of phosphorylated ERK1/2 did not change in HRGECs treated with control treatment, LIPUS, SonoVue, or the combination of LIPUS and SonoVue. In addition, the results of the cytotoxicity assays of HRGECs with pretreatment of PD98059 were similar for all the four treatments. Combination of LIPUS and SonoVue did not induce a decrease in cell proliferation (Figure 3(B), *p* = .773) and viability (Figure 3(C), *p* = .072), or an increase in apoptosis (Figure 3(D and E), *p* = .603). Take together, the cytotoxic effects of combined treatment of LIPUS and SonoVue are mediated by suppression of the ERK1/2 signaling pathway.

## Discussion

Ultrasound microbubbles have been found to enhance ultrasound cavitation effects and cause cellular apoptosis [24,25]. In this study, we examined the effect of LIPUS and SonoVue on cultured HRGECs. We found that LIPUS or SonoVue alone did not induce cytotoxicity of HRGECs, but the combination of the two treatments reduced cell proliferation and increases cell death. In addition, we found LIPUS combined with SonoVue decreased the phosphorylation of ERK1/2 in HRGECs. After pretreatment of PD98059, the inhibitor of ERK1/2 signaling pathway, the combined treatment of LIPUS and SonoVue did not induce additional cytotoxicity in HRGECs, suggesting that inhibition of the ERK1/2 signaling pathway mediates the cytotoxic effects.

LIPUS produces physical effects on cells and tissues, including microjet, radiation and microstreaming. These effects are often caused by acoustic cavitation [26]. In response to LIPUS, cells displayed distinct effects on different cell types. LIPUS promotes proliferation of human osteoblasts or synovial cells [6,19], but inhibits proliferation and induces apoptosis of human hapatocellular carcinoma cells [27]. Shindo et al. [28] found that LIPUS ameliorates the left ventricle remodeling after acute myocardial infarction in mice In addition, LIPUS does not affect chondrocyte in the cartilage, thus has no therapeutic potential in treating articular cartilage injury [29]. Similarly, LIPUS has no effect on metastatic bone tumors [30]. The effect of LIPUS on microvascular systems has not been reported. In this study, we found that LIPUS alone has not effected on HRGECs proliferation and cell death. However, LIPUS in combination with SonoVue induces significant cytotoxicity in HRGECs. SonoVue has been widely used as an ultrasound contrast agent. To date no cytotoxic effect of SonoVue has been reported. Ultrasound contrast agents may enhance ultrasonic cavitation by acting as a supply of cavitation nuclei [31,32]. In addition, the oscillation and collapse of microbubbles in the blood vessels might induce endothelial cell injury [33,34]. Application of LIPUS combined with microbubbles generates shear power, which might induce DNA degradation, causing the inhibition of cell proliferation [21]. As HRGECs form the primary layer of glomerular filtration, loss of HRGECs might induce hyper permeability and proteinuria. Thus, this study implied the potential risks of glomerular injuries by the combination of LIPUS and SonoVue.

MAPK signal cascades mediate the transmission of extracellular signals into cell nucleus and activate regulatory molecules in cytoplasm to modulate cellular functions [16,35]. The activation of the ERK1/2 signaling pathway involves in regulating cell proliferation, division, and maintenance of cellular skeleton [36]. Previous studies showed that LIPUS as a direct mechanical power may activate the ERK1/2 signaling pathway, leading to cell proliferation in the osteosarcoma cells and β1-integrin knock-out mouse fibroblasts [37,38]. Gao et al. [39] found that subsets of dental stem cell populations display increase cell proliferation via different MAPK modules in response to LIPUS. Dental pulp stem cell proliferation is specifically modulated by ERK1/2 inhibition, whereas JNK pathway is activated in bone marrow stem cells, and both JNK and p38 MAPK are activated in periodontal ligament stem cells [39]. In this study, LIPUS or SonoVue alone does not affect ERK1/2 pathway, but the combination of two treatments inhibits ERK1/2 activation. In addition, we found that ERK1/2 pathway mediates the cytotoxicity induced by LIPUS in combination with SonoVue. ERK1/2 pathway is essential for endothelial cell proliferation and survival [12,17]. Drugs and toxicants might induce cytotoxicity of endothelial cells via down-regulation of ERK1/2 pathway [17]. Taken together, this study identified a regulatory pathway which might be targeted to protect HRGECs from injuries from combination treatment of LIPUS and SonoVue.

In conclusion, LIPUS or SonoVue alone does not induce cytotoxicity, but the combined treatment of LIPUS and SonoVue decreases proliferation and increases cell death in HRGECs. The combined treatment inhibits the activation of ERK1/2 signaling pathway, which mediates the cytotoxic effects. Our study suggests that LIPUS in combination of SonoVue might induce cytotoxicity of glomerular endothelial cells, thus potentially affect glomerular filtration in the kidney.
